# Supplementary material for: Mitochondrial genomes of three Tetrigoidea species and phylogeny of Tetrigoidea
Source: PeerJ. 2017 Nov 15;5:e4002. doi: 10.7717/peerj.4002 (PMC5694214; doi:10.7717/peerj.4002)
Supplement: Table S1 [file peerj-05-4002-s001.doc]

**Table S1 Primer pairs used in PCR amplification of the mitogenomes of *Formosatettix qinlingensis*, *Coptotettix longjiangensis* and *Thoradonta obtusilobata*.**

| Fragment | Primer F | Sequence (5'→3') | Primer R | Sequence (5'→3') |
| --- | --- | --- | --- | --- |
| T1 | TF34 | GCCTGATAAAAAGGRTTAYYTTGATA | TR1284 | ACARCTTTGAAGGYTAWTAGTTT |
|  | TF210 | AATTAAGCTACTAGGTTCATACCC |  |  |
| T2 | TF944 | GGACTACCACCATTWHTWGG | TR2072 | GGGTCAAAGAATGAWGTATT |
| T3 | LP03t | CATTTATTTTGATTYTTTGGWCAYCCAGAAGT | LP06t | TGATTAGCTCCACAAATTTCTGAACATTGACC |
| AT01 | TCOBU | TYTCAACAAAYCAYAARGATATTGG | TCOBL | TAAACTTCWGGRTGWCCAAARAATCA |
| AT02 | TF1652 | GGAGGATTYGGAAATTGATTAGT | TR2289 | TACTGTAAATATATGRTGDGCTC |
| T4 | TF3553 | ATTGAYGCAACACCMGGACG | TR7065 | CAGTAATACGCCTCTYTTTG |
|  | TF4292 | GTTGATTATAGACCWTGRCC |  |  |
|  | TF4970 | GCAGCTGCYTGATAYTGRCA |  |  |
| AT03 | TF2899 | ACAATTGGTCAYCAATGATAYTG | TR4052 | ATGTCCWGCAATYATATTWGC |
| T5 | TF6065 | AGAGAGGCGTATTACTGTTA | TR7306 | TTTRAAGGATTCTCAGGATA |
|  | TF5247 | CCATTTGAATGTGGRTTTGAYCC |  |  |
| T6 | TF7065 | GAMACAARACCTAACCCATCYCA | TR8653 | TGAGGTTATCAACCNGARCG |
| T7 | TF8572 | CGCTCAGGYTGRTACCCYCA | LP04t | AAAATWGCRTAWGCAAATARAAAATATCAT |
|  | TF9148 | ACCTAAAGCTCCCTCACAWAC | TR10510 | TATCTACAGCRAATCCYCCYCA |
| T8 | LP05t | WACACCAGTTCATATTCAACCAGAATGATATT | TR14108 | TACACATCGCCCGTCRCTCT |
|  |  |  | TR13288 | CGCCTGTTTAACAAAAACAT |
| AT04 | 16SF | CGCCTGTTTATCAAAAACAT | 16SR | CTCCGGTTTGAACTCAGATCA |
| T9 | TF12388 | CCGGTTTGAACTCARATCATGTAA | TR14245 | GTGCCAGCAGYYGCGGTTANAC |
| T10 | TF13697 | GTACAYCTACTATGTTACGACTT | TR18 | TCCTATCAARRTAAYCCTTT |
|  | TF14110 | ATAATAGGGTATCTAATCCTAGT | TR993 | GGTAAAAATCCTAAAAATGGNGG |
|  | TF14639 | GCGGCTGCTGGCACGAAA | TR947 | AATCCTAGAAATGGTGGYAR |
| clone | M13-47 | CGCCAGGGTTTTCCCAGTCACGAC | RV-M | GAGCGGATAACAATTTCACACAGG |
