# Supplementary material for: Mitochondrial genomes of three Tetrigoidea species and phylogeny of Tetrigoidea
Source: PeerJ. 2017 Nov 15;5:e4002. doi: 10.7717/peerj.4002 (PMC5694214; doi:10.7717/peerj.4002)
Supplement: Table S2 [file peerj-05-4002-s002.doc]

**Table S2 Taxon samples, mitochondrial genome sequence accession numbers, and representative families and subfamilies following the classification of Otte.**

| Suborder | Superfamily | Family | Subfamily | Species | Accession number |
| --- | --- | --- | --- | --- | --- |
| Caelifera | Acridoidea | Acrididae | Thrinchinae | *Pseudotmethis rubimarginis* | JX468878 |
|  |  | Acrididae | Thrinchinae | *Filchnerella helanshanensis* | JX468877 |
|  |  | Pyrgomorphidae | Pyrgomorphinae | *Mekongiella xizangensis* | HM583654 |
|  |  | Pyrgomorphidae | Pyrgomorphinae | *Atractomorpha sinensis* | EU263919 |
|  |  | Acrididae | Catantopinae | *Traulia szetschuanensis* | EU914849 |
|  |  | Acrididae | Oxyinae | *Oxya chinensis* | EF437157 |
|  |  | Acrididae | Melanopline | *Prumna arctica* | GU294758 |
|  |  | Acrididae | Calliptaminae | *Calliptamus italicus* | EU938373 |
|  |  | Acrididae | Oedipodinae | *Locusta migratoria* | X80245 |
|  |  | Acrididae | Gomphocerinae | *Arcyptera coreana* | GU324311 |
|  |  | Acrididae | Oedipodinae | *Ceracris kiangsu* | GU270284 |
|  |  | Acrididae | Acridinae | *Acrida cinerea* | GU344100 |
|  |  | Acrididae | Gomphocerinae | *Gomphocerus sibiricus* | JX122541 |
|  | Eumastacoidea | Episactidae | Episactinae | *Pielomastax zhengi* | JF411955 |
|  | Tetrigoidea | Tetrigidae | Cladonotinae | *Trachytettix bufo* | JX913766 |
|  |  | Tetrigidae | Tetriginae | *Tetrix japonica* | JQ340002 |
|  |  | Tetrigidae | Tetriginae | *Alulatettix yunnanensis* | JQ272702 |
|  |  | Tetrigidae | Tetriginae | *Formosatettix qinlingensis* | KY798412 |
|  |  | Tetrigidae | Tetriginae | *Coptotettix longjiangensis* | KY798413 |
|  |  | Tetrigidae | Scelimeninae | *Thoradonta obtusilobata* | KY798414 |
|  | Tridactyloidea | Tridactylidae | Tridactylinae | *Ellipes minuta* | GU945502 |
| Ensifera | Tettigonioidea | Tettigoniidae | Mecopodinae | *Mecopoda niponensis* | JQ917909 |
|  | Grylloidea | Gryllidae | Myrmecophilinae | *Myrmecophilus manni* | EU938370 |
|  | Gryllotalpoidea | Gryllotalpidae | Gryllinae | *Gryllotalpa orientalis* | AY660929 |
