# Supplementary material for: Mitochondrial genomes of three Tetrigoidea species and phylogeny of Tetrigoidea
Source: PeerJ. 2017 Nov 15;5:e4002. doi: 10.7717/peerj.4002 (PMC5694214; doi:10.7717/peerj.4002)
Supplement: Table S3 [file peerj-05-4002-s003.doc]

**Table S3 The values of A+T content, AT-skew, G+C content and GC-skew in 21 Caelifera mitogenomes.**

| Species | A+T content (%) | AT-skew | G+C content (%) | GC-skew |
| --- | --- | --- | --- | --- |
| *Acrida cinerea* | 76.07 | 0.17 | 23.93 | -0.16 |
| *Alulatettix yunnanensis* | 75.24 | 0.19 | 24.76 | -0.21 |
| *Arcyptera coreana* | 76.34 | 0.13 | 23.66 | -0.13 |
| *Atractomorpha sinensis* | 74.29 | 0.16 | 25.71 | -0.18 |
| *Calliptamus italicus* | 73.26 | 0.14 | 26.74 | -0.16 |
| *Ceracris kiangsu* | 74.90 | 0.16 | 25.10 | -0.17 |
| *Ellipes minuta* | 66.48 | 0.01 | 33.52 | -0.30 |
| *Filchnerella helanshanensis* | 72.44 | 0.16 | 27.56 | -0.19 |
| *Gomphocerus sibiricus* | 74.88 | 0.13 | 25.12 | -0.13 |
| *Locusta migratoria* | 75.33 | 0.18 | 24.67 | -0.18 |
| *Mekongiella xizangensis* | 73.55 | 0.16 | 26.45 | -0.21 |
| *Oxya chinensis* | 75.89 | 0.12 | 24.11 | -0.13 |
| *Pielomastax zhengi* | 71.77 | 0.10 | 28.23 | -0.11 |
| *Prumna arctica* | 76.07 | 0.13 | 23.93 | -0.14 |
| *Pseudotmethis rubimarginis* | 72.36 | 0.16 | 27.64 | -0.19 |
| *Tetrix japonica* | 75.57 | 0.19 | 24.43 | -0.21 |
| *Trachytettix bufo* | 71.64 | 0.21 | 28.36 | -0.25 |
| *Traulia szetschuanensis* | 74.55 | 0.14 | 25.45 | -0.16 |
| *Formosatettix qinlingensis* | 75.56 | 0.19 | 24.44 | -0.21 |
| *Coptotettix longjiangensis* | 73.09 | 0.22 | 26.91 | -0.23 |
| *Thoradonta obtusilobata* | 71.87 | 0.14 | 28.13 | -0.28 |
