# Supplementary material for: Mitochondrial genomes of three Tetrigoidea species and phylogeny of Tetrigoidea
Source: PeerJ. 2017 Nov 15;5:e4002. doi: 10.7717/peerj.4002 (PMC5694214; doi:10.7717/peerj.4002)
Supplement: Table S4 — Note: The highest codon usages within relative synonymous codons are in bold, with codons corresponded to tRNA anticodons written in green, and not corresponded to tRNA anticodons in red. [file peerj-05-4002-s004.doc]

**Table S4 The relative synonymous codon usage (RSCU) of 21 Caelifera mitochondrial PCGs.** Note: The highest codon usages within relative synonymous codons are in bold, with codons corresponded to tRNA anticodons written in green, and not corresponded to tRNA anticodons in red.

| Codon | n(RSCU) | Codon | n(RSCU) | Codon | n(RSCU) | Codon | n(RSCU) |
| --- | --- | --- | --- | --- | --- | --- | --- |
| **UUU(F)** | **272.8(1.61)** | UCU(S) | 106.6(2.33) | **UAU(Y)** | **142.4(1.67)** | **UGU(C)** | **38.4(1.76)** |
| UUC(F) | 66.6(0.39) | UCC(S) | 15.9(0.35) | UAC(Y) | 28.2(0.33) | UGC(C) | 5.3(0.24) |
| **UUA(L)** | **326.4(3.72)** | **UCA(S)** | **117.5(2.57)** | UAA(*) | 0.0(0.00) | **UGA(W)** | **85.3(1.72)** |
| UUG(L) | 54.7(0.62) | UCG(S) | 6.7(0.15) | UAG(*) | 0.0(0.00) | UGG(W) | 13.9(0.28) |
| CUU(L) | 60.7(0.69) | CCU(P) | 56.7(1.69) | **CAU(H)** | **50.3(1.44)** | CGU(R) | 20.0(1.47) |
| CUC(L) | 9.8(0.11) | CCC(P) | 12.6(0.37) | CAC(H) | 19.5(0.56) | CGC(R) | 1.3(0.10) |
| **CUA(L)** | **67.5(0.77)** | **CCA(P)** | **60.9(1.81)** | **CAA(Q)** | **56.9(1.73)** | **CGA(R)** | **30.1(2.21)** |
| CUG(L) | 7.4(0.08) | CCG(P) | 4.1(0.12) | CAG(Q) | 9.0(0.27) | CGG(R) | 3.1(0.23) |
| **AUU(I)** | **315.1(1.68)** | ACU(T) | 60.9(1.24) | **AAU(N)** | **138.0(1.58)** | AGU(S) | 31.5(0.69) |
| AUC(I) | 59.8(0.32) | ACC(T) | 22.3(0.45) | AAC(N) | 36.8(0.42) | AGC(S) | 4.0(0.09) |
| **AUA(M)** | **233.2(1.68)** | **ACA(T)** | **109.6(2.23)** | **AAA(K)** | **77.4(1.54)** | **AGA(S)** | **78.7(1.72)** |
| AUG(M) | 44.0(0.32) | ACG(T) | 4.0(0.08) | AAG(K) | 23.0(0.46) | AGG(S) | 4.6(0.10) |
| **GUU(V)** | **91.5(1.99)** | GCU(A) | 65.4(1.66) | **GAU(D)** | **62.1(1.64)** | GGU(G) | 86.3(1.59) |
| GUC(V) | 7.6(0.16) | GCC(A) | 17.0(0.43) | GAC(D) | 13.7(0.36) | GGC(G) | 7.5(0.14) |
| GUA(V) | 75.4(1.64) | **GCA(A)** | **72.3(1.83)** | **GAA(E)** | **67.2(1.65)** | **GGA(G)** | **99.2(1.82)** |
| GUG(V) | 9.4(0.20) | GCG(A) | 3.0(0.08) | GAG(E) | 14.2(0.35) | GGG(G) | 24.6(0.45) |
