# Supplementary material for: Mitochondrial genomes of three Tetrigoidea species and phylogeny of Tetrigoidea
Source: PeerJ. 2017 Nov 15;5:e4002. doi: 10.7717/peerj.4002 (PMC5694214; doi:10.7717/peerj.4002)
Supplement: Table S5 [file peerj-05-4002-s005.doc]

**Table S5 The average ratio of Ka/Ks and G+C content for each PCG of six Tetrigidae mitogenomes.**

| Gene | Ka/Ks value | G+C content (%) |
| --- | --- | --- |
| nad2 | 0.27 | 24.06 |
| cox1 | 0.07 | 32.73 |
| cox2 | 0.09 | 29.30 |
| atp8 | 0.82 | 18.78 |
| atp6 | 0.22 | 26.30 |
| cox3 | 0.16 | 32.30 |
| nad3 | 0.18 | 26.45 |
| nad5 | 0.26 | 24.70 |
| nad4 | 0.23 | 24.97 |
| nad4L | 0.41 | 22.18 |
| nad6 | 0.37 | 20.43 |
| cytb | 0.13 | 30.16 |
| nad1 | 0.21 | 26.87 |
