# Supplementary figures and images for: Mitochondrial genomes of three Tetrigoidea species and phylogeny of Tetrigoidea
Source: PeerJ. 2017 Nov 15;5:e4002. doi: 10.7717/peerj.4002 (PMC5694214; doi:10.7717/peerj.4002)

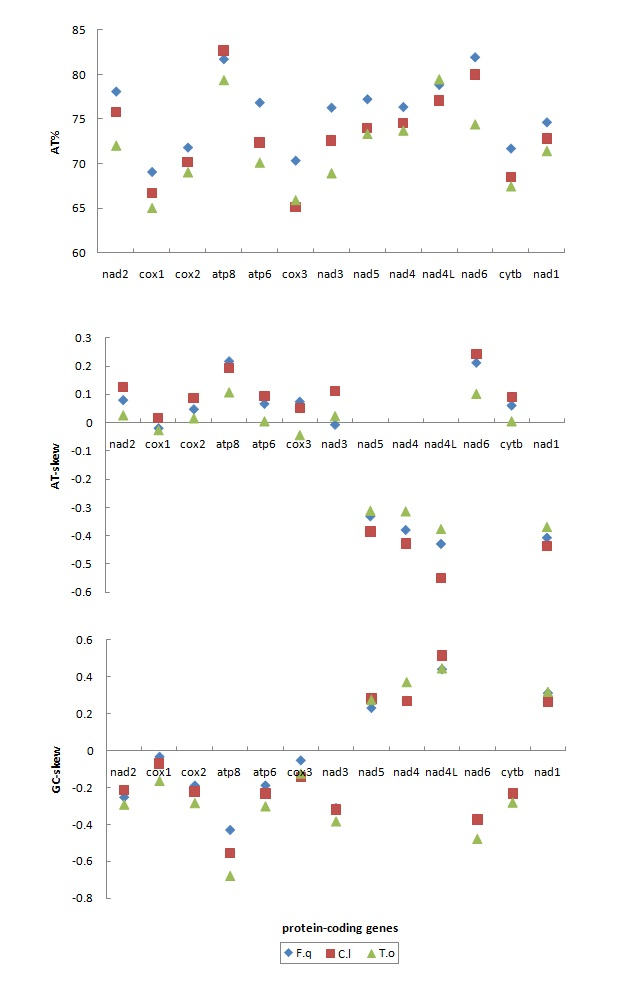

Supplement: Figure S1 — Notes: Formosatettix qinlingensis (F. q), Coptotettix longjiangensis (C. l) and Thoradonta obtusilobata (T. o). [file peerj-05-4002-s006.tif]

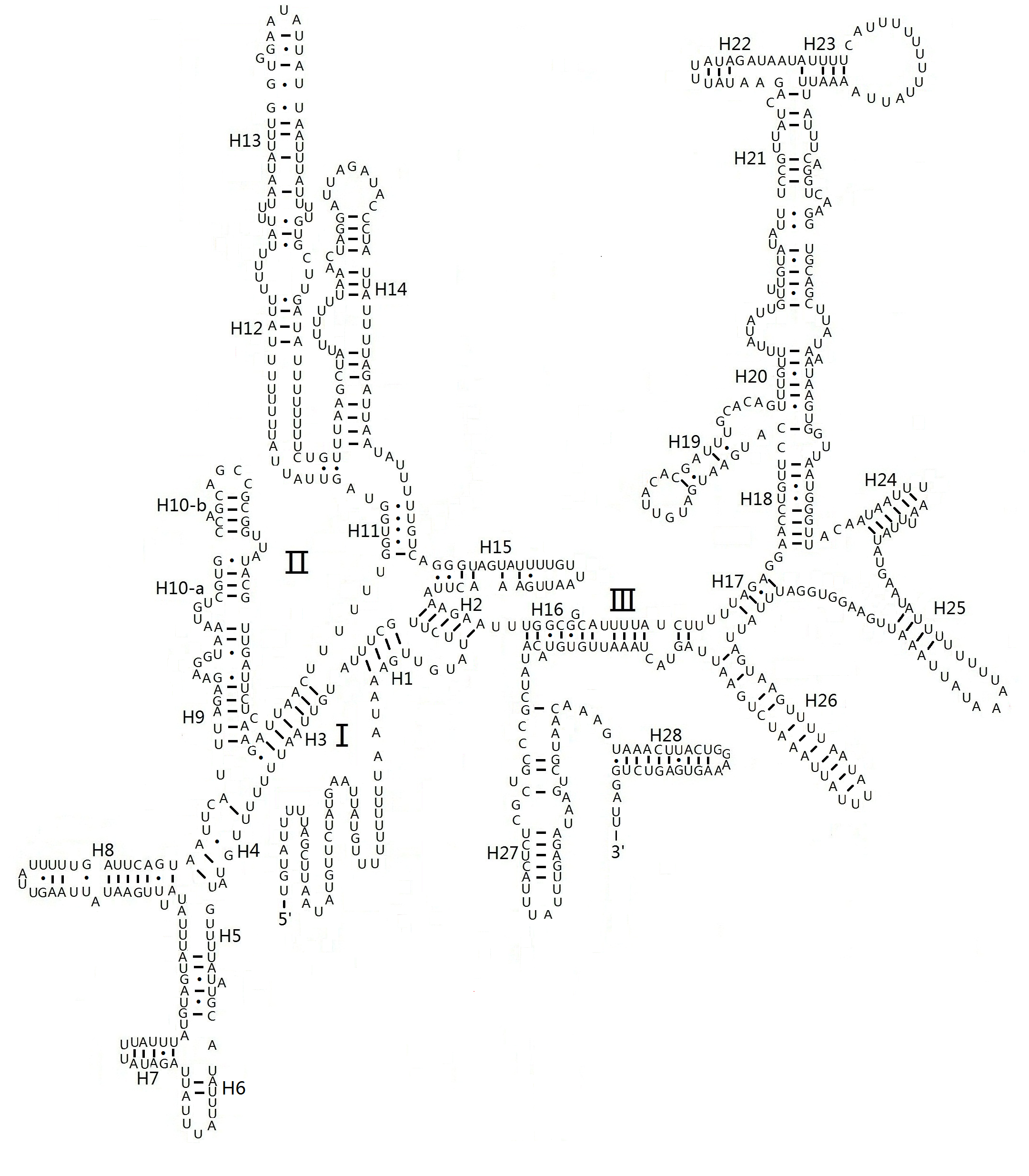

Supplement: Figure S2 — Notes: Each helix is numbered progressively from the 5′ to the 3′ end. Domains are labelled with Roman numerals. [file peerj-05-4002-s007.tif]

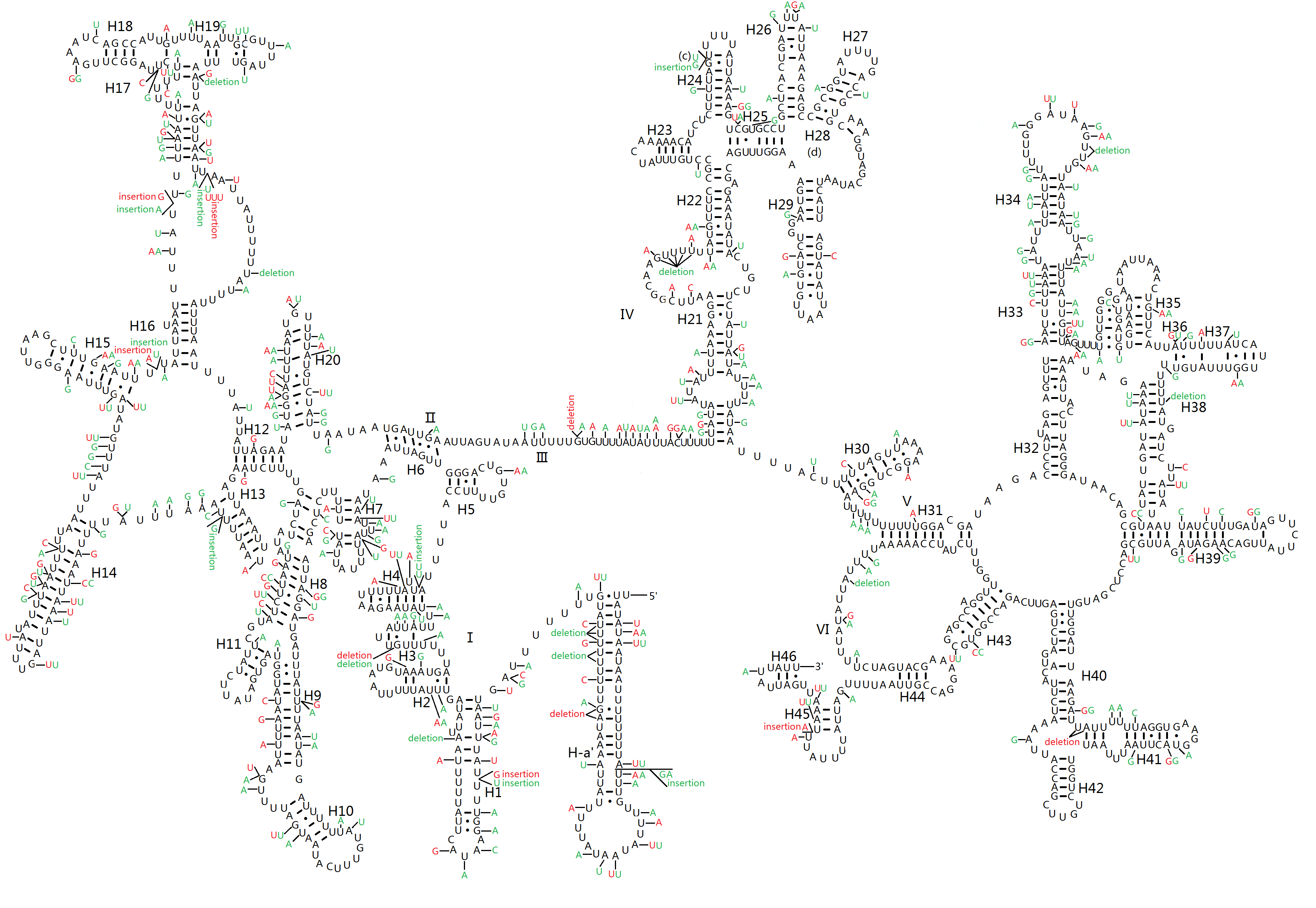

Supplement: Figure S3 — Notes: Each helix is numbered progressively from the 5′ to the 3′ end. Domains are labelled with Roman numerals. Single variable sites from two species are labelled in different colours (Coptotettix longjiangensis: red; Thoradonta obtusilobata: green). [file peerj-05-4002-s008.tif]

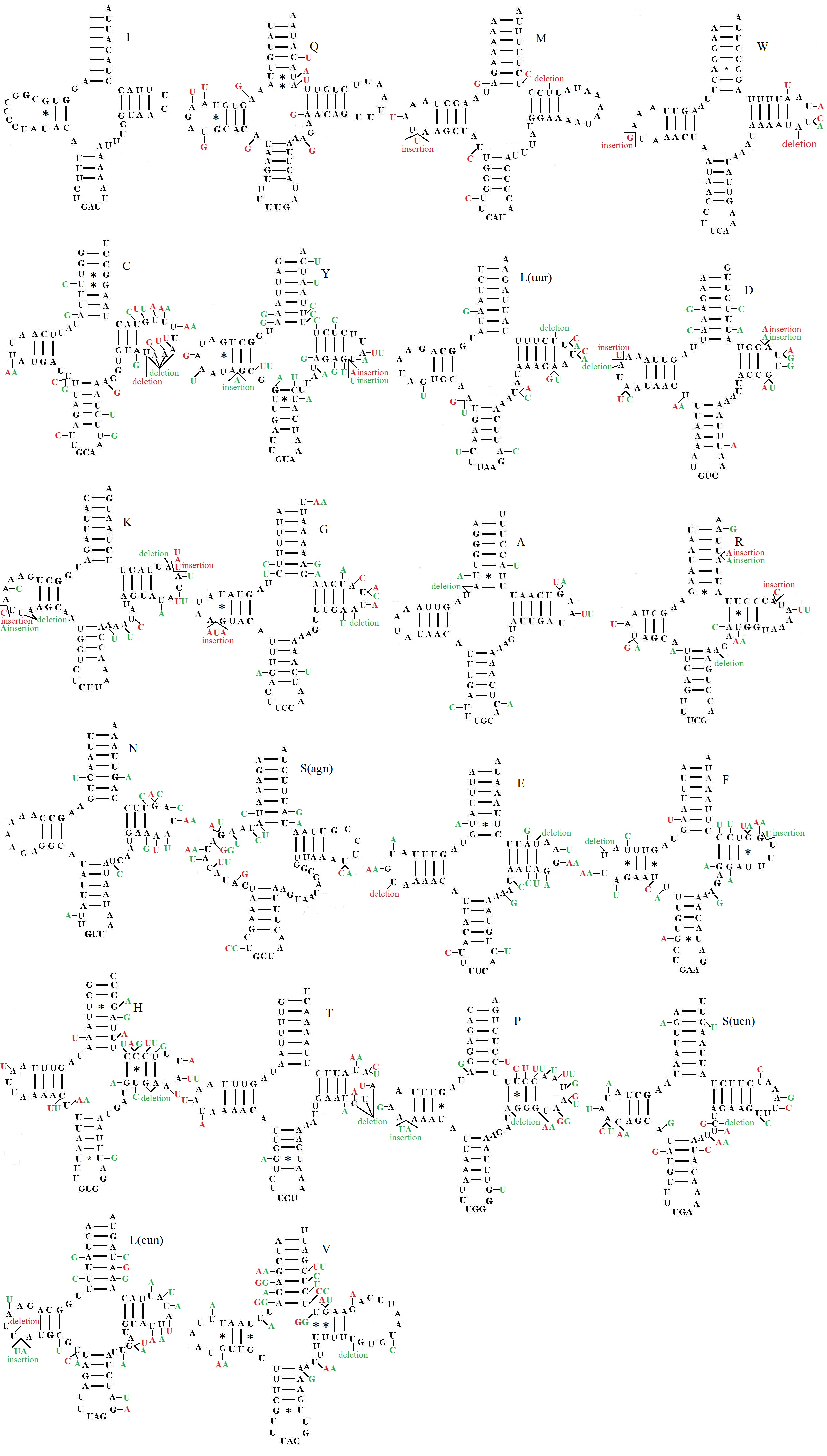

Supplement: Figure S4 — Notes: Single variable sites from two species are labelled in different colours (Coptotettix longjiangensis: red; Thoradonta obtusilobata: green). [file peerj-05-4002-s009.tif]

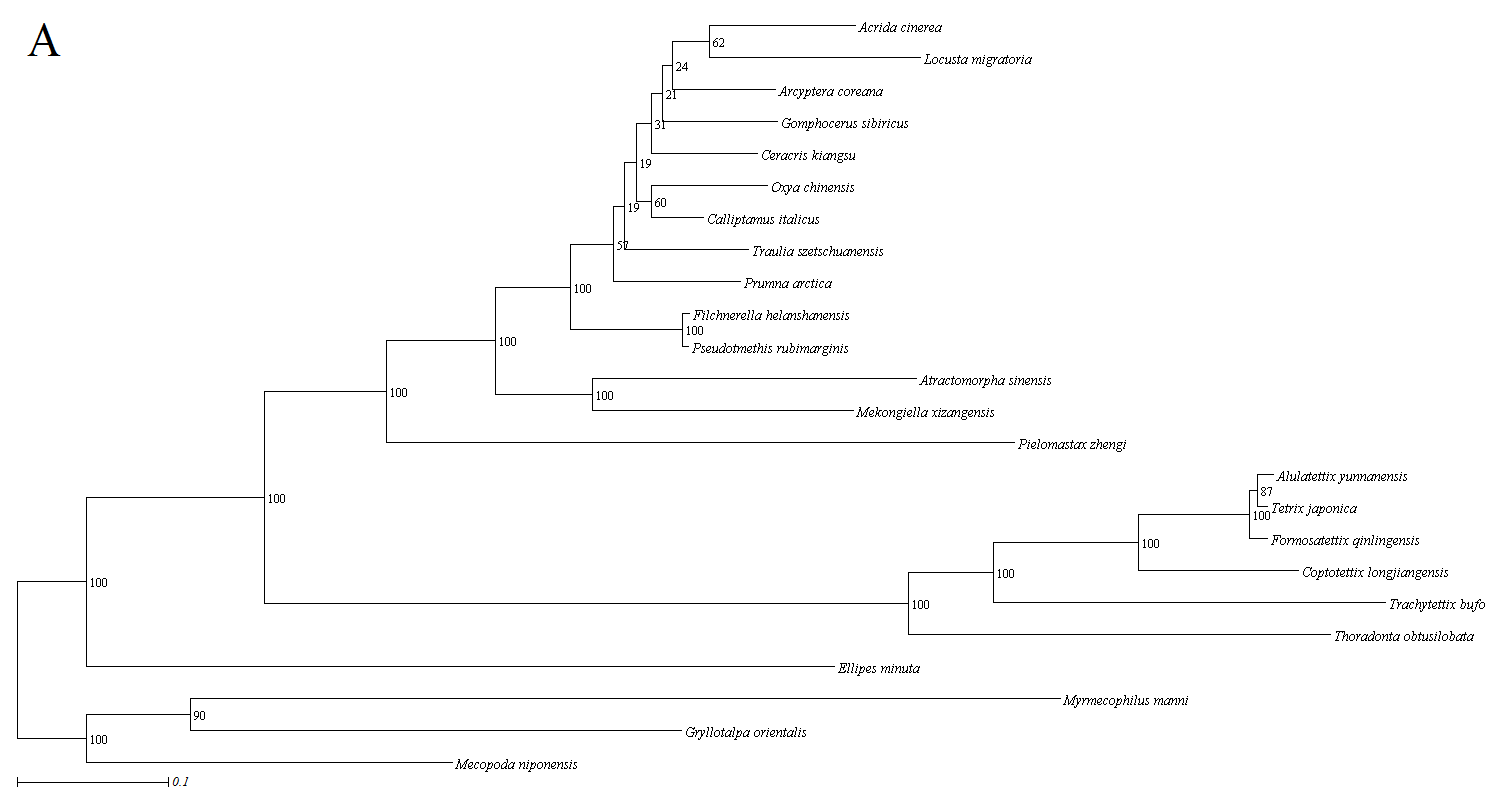


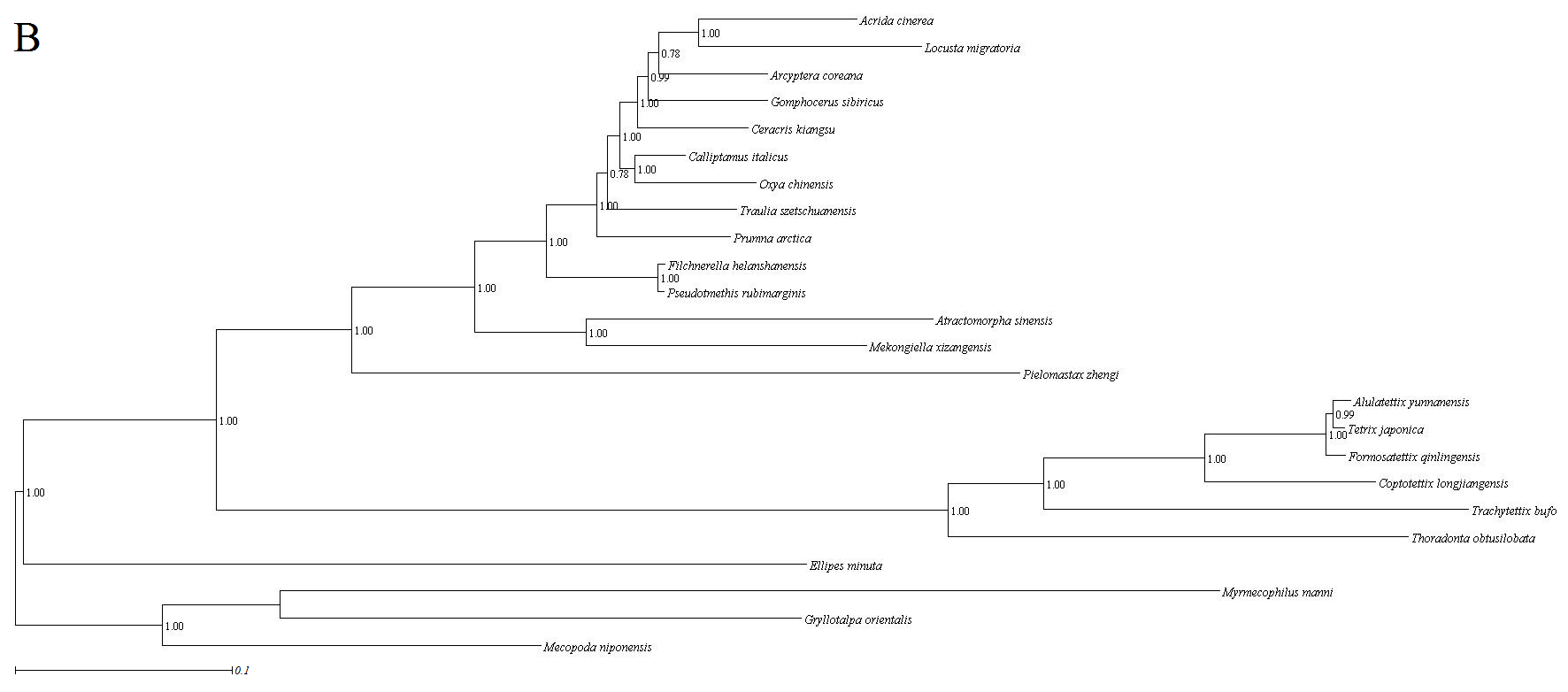


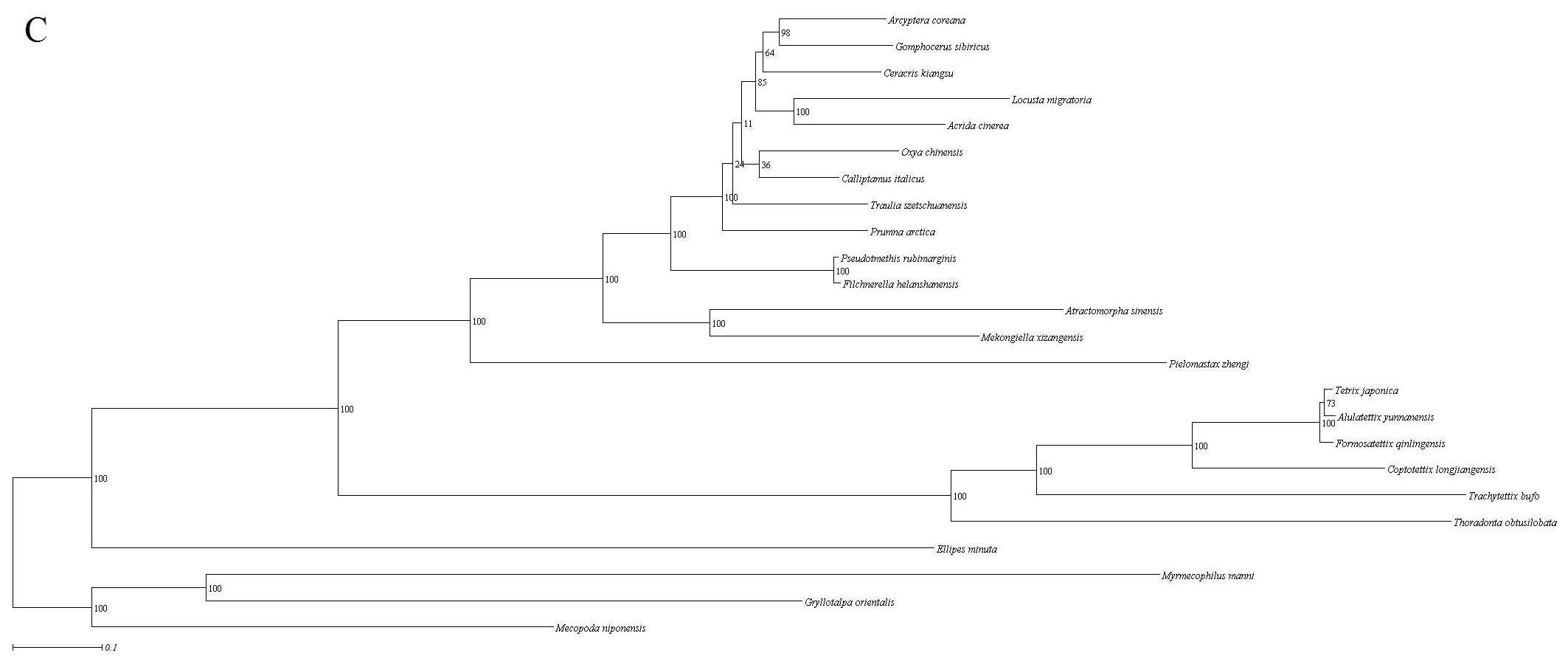


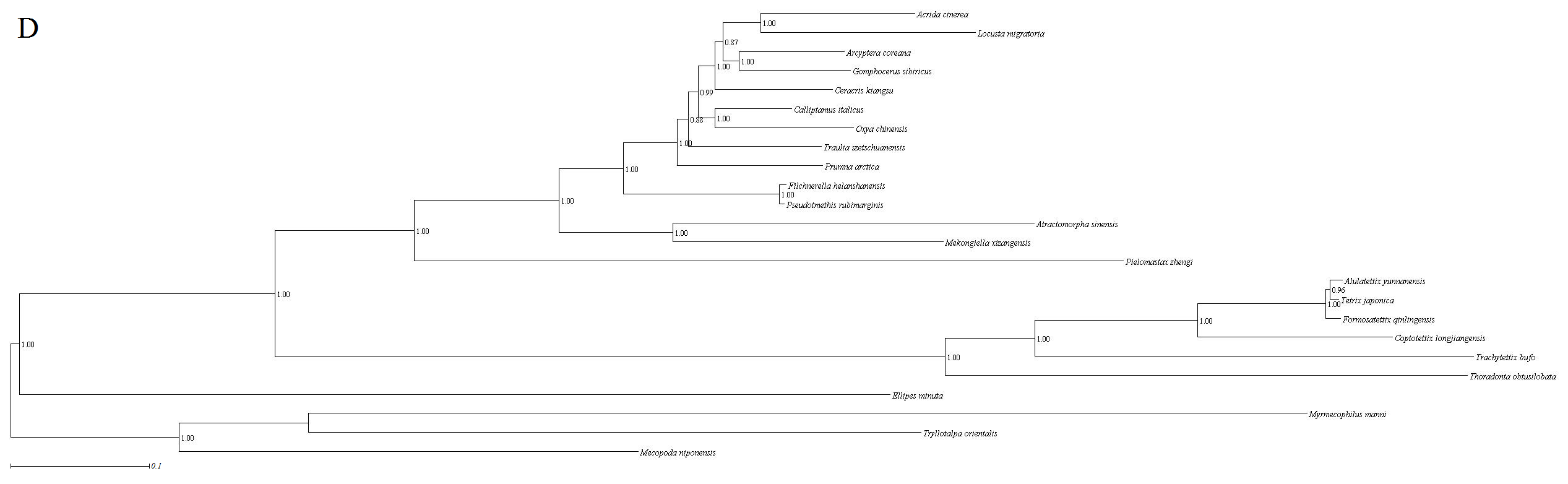


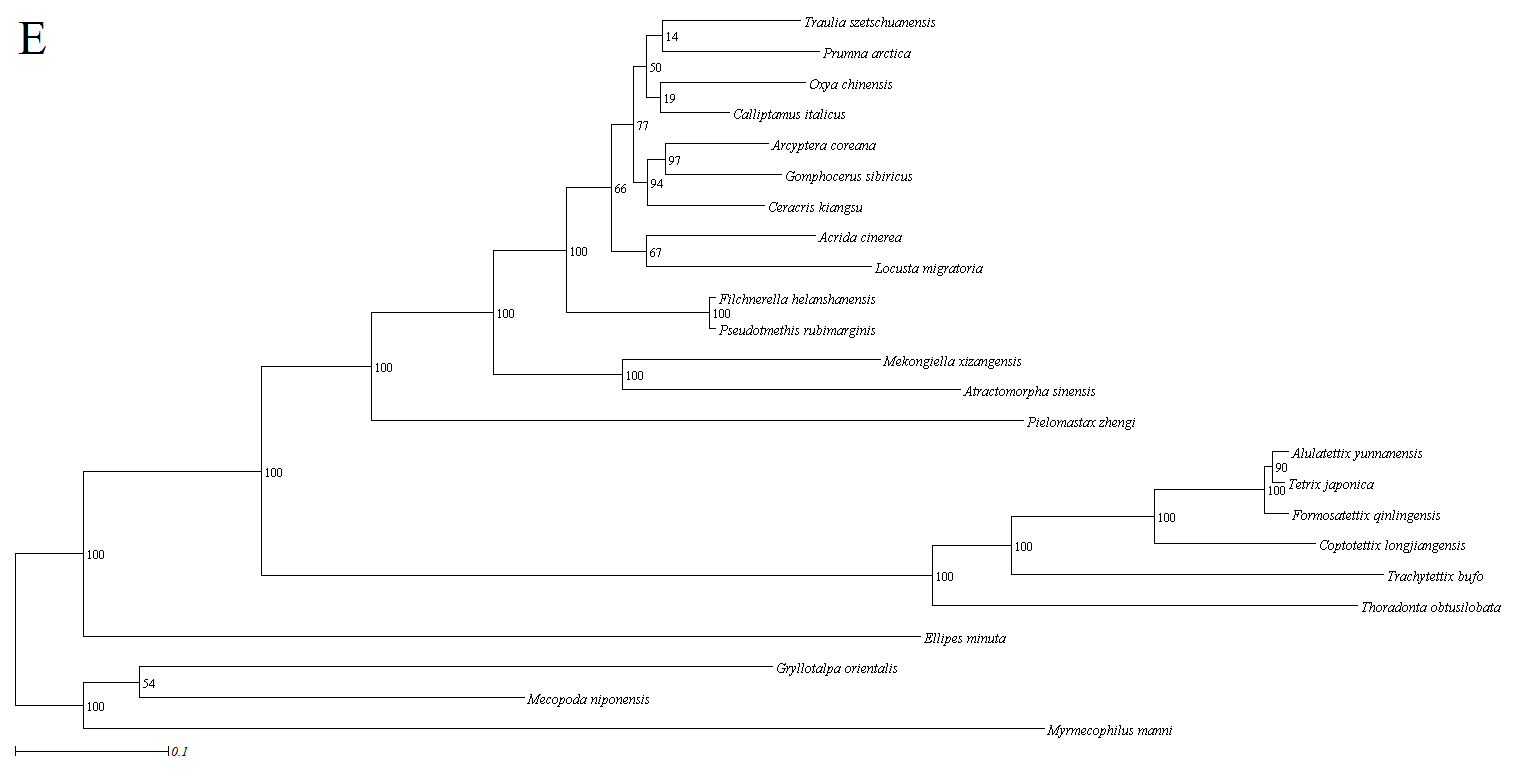


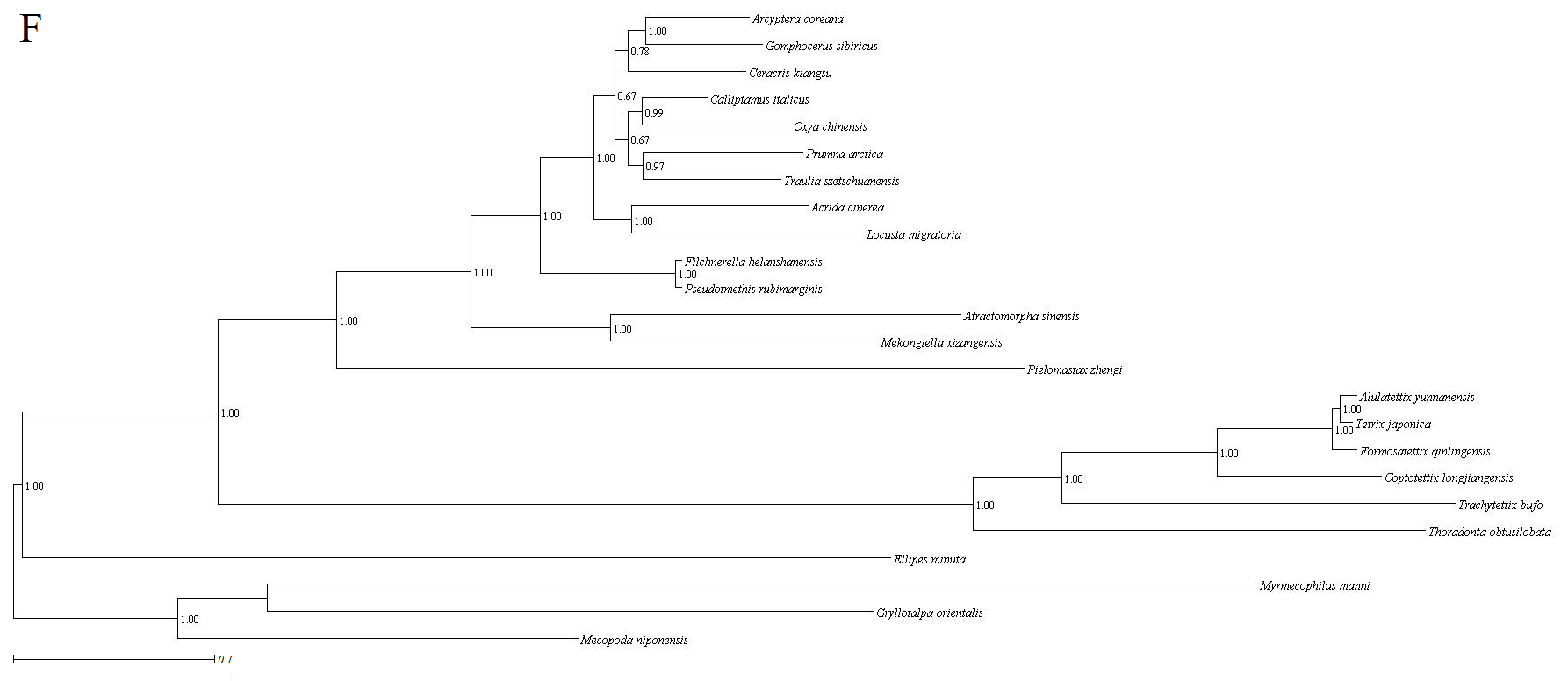


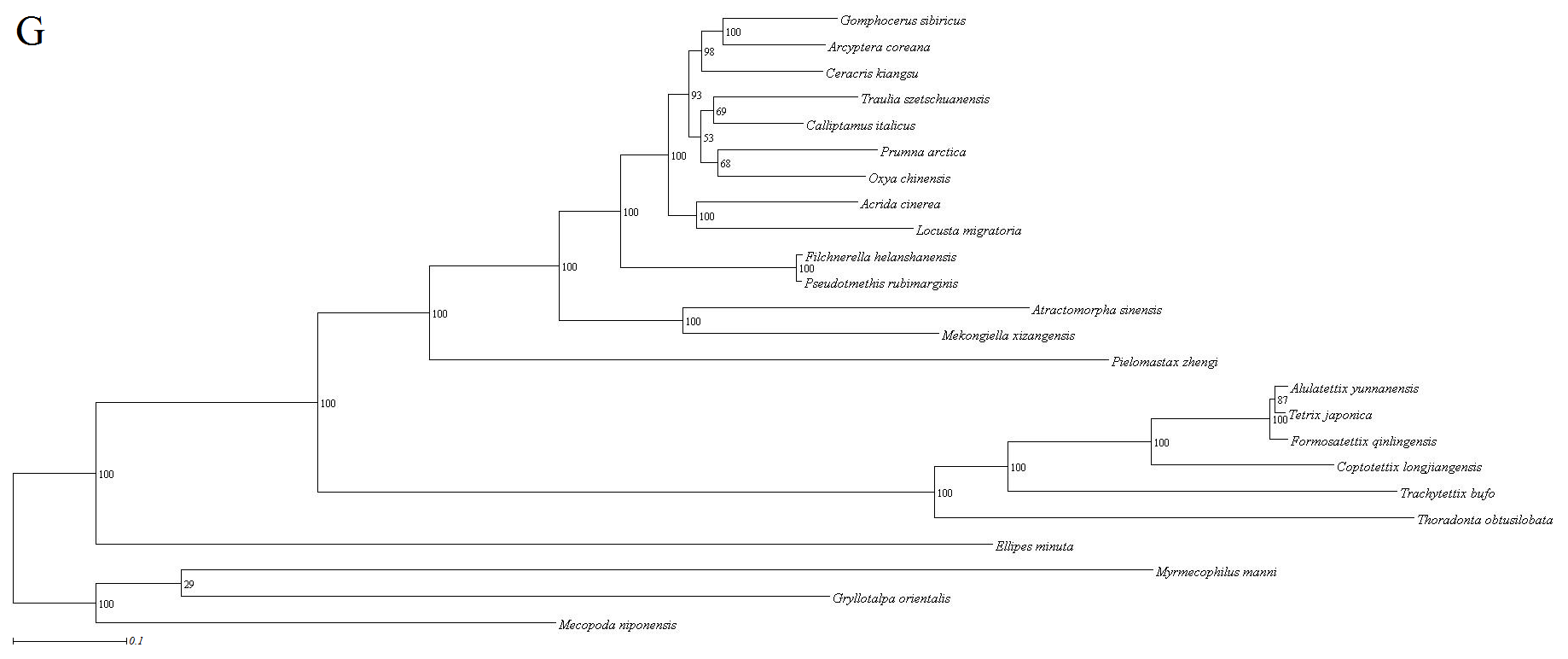


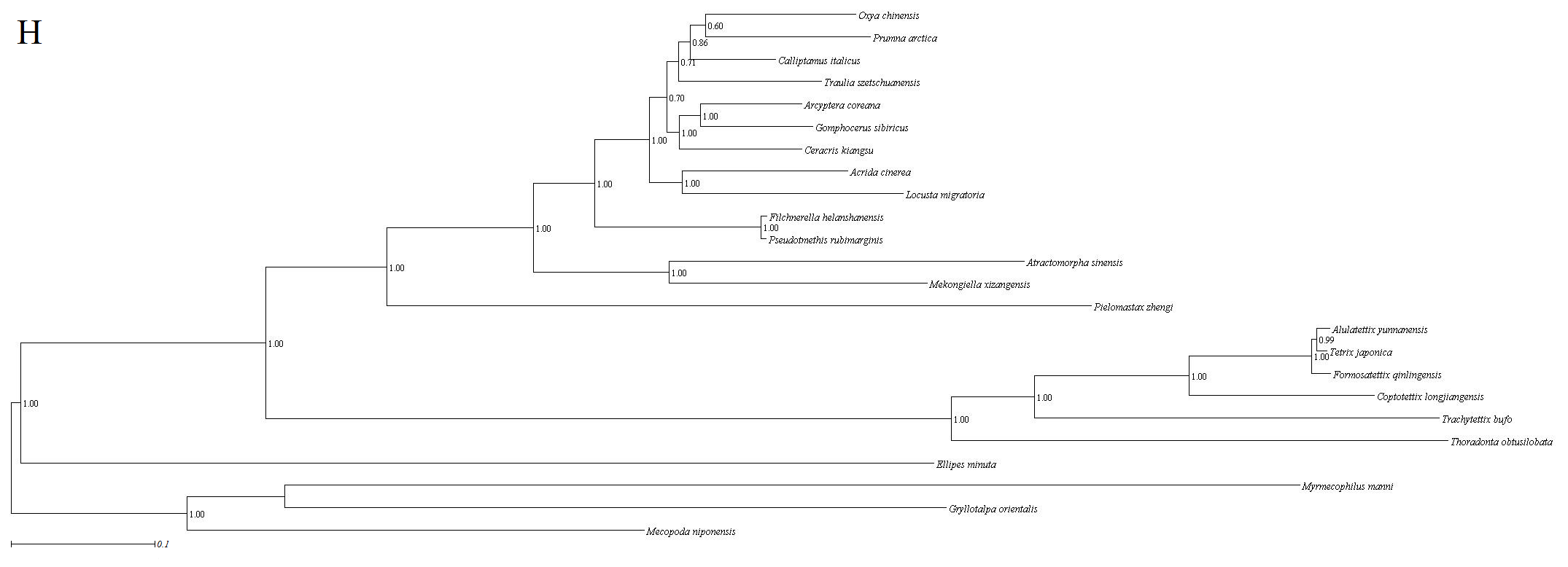

Supplement: Figure S5 — Notes: (A) PCG12-ML tree, (B) PCG12-BI tree, (C) PCG123RY-ML tree, (D) PCG123RY-BI tree, (E) PCG12rRNA-ML tree, (F) PCG12 rRNA-BI tree, (G) PCG123RYrRNA-ML tree, (H) PCG123RYrRNA-BI tree. [file peerj-05-4002-s010.doc]
